# Modified PCR protocol to increase sensitivity for determination of bacterial community composition

**DOI:** 10.1186/s40168-020-00958-y

**Published:** 2021-04-13

**Authors:** Kayla M. Williamson, Brandie D. Wagner, Charles E. Robertson, Mark J. Stevens, Marci K. Sontag, Peter M. Mourani, J. Kirk Harris

**Affiliations:** 1 Department of Biostatistics and Informatics, Colorado School of Public Health, University of Colorado School of Medicine, 13001 17th Place, Mail Stop B119, Aurora, CO 80045 USA; 2 Section of Critical Care, Department of Pediatrics, University of Colorado School of Medicine and Children’s Hospital Colorado, 13123 E. 16th Ave. Box B395, Aurora, CO 80045 USA; 3 Division of Infectious Diseases, School of Medicine, University of Colorado, 12700 East 19th Avenue, Mail Stop B168, Aurora, CO 80045 USA; 4 Department of Epidemiology, Colorado School of Public Health, University of Colorado School of Medicine, 13001 17th Place, Mail Stop B119, Aurora, CO 80045 USA

**Keywords:** 16S rRNA gene, Microbiome, Amplicon, Mechanical ventilation

## Abstract

**Background:**

The objective of this project was to increase the sensitivity of sequence-based bacterial community determination without impacting community composition or interfering with cluster formation during sequencing. Two PCR protocols (standard and modified) were examined in airway samples where we observed a large range in bacterial load (3.1–6.2 log_10_ 16S rRNA gene copies/reaction). Tracheal aspirate (TA) samples (*n* = 99) were collected from sixteen children requiring mechanical ventilation at a single center. DNA was extracted, and total bacterial load (TBL) was assessed using qPCR. Amplification of 16S rRNA was attempted with both protocols in all samples.

**Results:**

PCR product was observed using both protocols in 52 samples and in 24 additional samples only with the modified protocol. TBL, diversity metrics, and prominent taxa were compared for samples in three groups based on success of the two protocols (successful with both, success with modified only, unsuccessful for both). TBL differed significantly across the three groups (*p*<0.001). Specifically, the modified protocol allowed amplification from samples with intermediate TBL. Shannon diversity was similar between the two protocols, and Morisita-Horn beta diversity index showed high agreement between the two protocols within samples (median value 0.9997, range 0.9947 to 1). We show that both protocols identify similar communities, and the technical variability of both protocols was very low. The use of limited PCR cycles was a key feature to limit impact of background by exclusion of 24% of samples with no evidence of bacterial DNA present in the sample.

**Conclusion:**

The modified amplification protocol represents a viable approach that increased sensitivity of bacterial community analysis, which is important for study of the human airway microbiome where bacterial load is highly variable.

**Video abstract**

**Supplementary Information:**

The online version contains supplementary material available at 10.1186/s40168-020-00958-y.

## Introduction

High-throughput sequencing allows parallel examination of bacterial communities from large numbers of samples. This is accomplished by using indexed primers to tag individual samples with a short DNA index sequence that allows assignment of each sequence to the sample of origin. High-throughput sequencing platforms require additional extension of the indexed primers with oligonucleotides to facilitate sequencing. The additional primer oligonucleotides required for high-throughput sequencing impacts the efficiency of amplification. This is problematic for samples with low levels of bacterial DNA or in cases where large amounts of non-target DNA are present, which is common in clinical specimens. We utilized a qPCR-based approach to estimate the bacterial load [1, 2] rather than total DNA concentration to perform sequencing amplification. The bacterial load determined by qPCR is more informative than DNA concentration regarding the amount of bacteria present in clinical samples where the majority of DNA is non-bacterial.

To reduce the impact of background DNA on bacterial community composition limiting PCR cycle numbers is important [3]. We utilize a conservative approach that eliminates samples from analysis due to inability to amplify adequate PCR product in order to decrease the contribution of background DNA in sequencing projects. This is accomplished by validating the background level in each tube of PCR reagent by running positive and negative amplification controls prior to use with clinical samples. Amplification is limited to a cycle number below this tube-specific background (never exceeding 30 cycles), which limits the amount of background contribution to the sequencing experiments. We observed poor sensitivity with our standard amplification protocol in association with low bacterial load in human airway samples. To address this poor sensitivity, we designed a modified amplification protocol that incorporated primers specific to the target region without sequencing adapters.

The goal of the experimental modification was to increase the sensitivity of bacterial community analysis for samples containing low bacterial load with the expectation that bacterial community identification can be informative to underlying pathobiology of patients, even in low load settings. To test this idea, we utilized tracheal aspirate samples from children requiring invasive mechanical ventilation support that contained a mixture of high and low bacterial load DNA extractions. DNA amplification efficiency was tested using both protocols. To examine technical variation, we utilized samples with adequate load to run replicate libraries using both protocols.

## Methods

### Samples

Tracheal aspirate (TA) samples were collected from children requiring invasive mechanical ventilation at a single center under an IRB-approved protocol (COMIRB 14-1530). Written informed consent and HIPPA authorization were obtained from all subjects or their legal guardians if <18 years of age. The initial sample was collected within 24 h of initial intubation, and subsequent samples were collected daily for up to 14 days. Samples were collected during standard of care endotracheal tube clearance into a sterile mucous trap via in-line suctioning equipment. Depth of suctioning was standardized by protocol. Per care guidelines up to 0.5 mL of non-bacteriostatic sterile saline could be used to clear tubing and facilitate collection. Specimens were transferred from the sterile mucous trap to 2 mL cryovials, within 5 min of collection, and stored at −80 °C. Each sample was assigned an alphanumeric identifier that was utilized throughout the laboratory processing.

### DNA extraction and determination of bacterial load

DNA was produced using the Qiagen EZ1 Advanced extraction platform. The frozen tracheal aspirate was thawed at 4 °C, and a 200 μl aliquot was transferred into the tube supplied with the EZ1 DNA Tissue Kit (Qiagen). DNA extraction controls were generated using 200 μl of DEPC-treated water (same used for PCR) in parallel with samples. Extraction was performed using manufacturer’s instructions, and DNA was eluted in 100 μl of Tris/EDTA (TE, supplied in the cartridge). Each DNA was diluted 1:40 in TE (to preserve DNA sample volume), and 4 μl of the dilution (dilution factor of 10) was used in triplicate reactions to determine total bacterial load based on the assay published by [2]. Briefly, this assay is based on TaqMAN qPCR approach and utilizes three conserved regions within the 16S rRNA gene (338/805 regions for primers, 515 region for probe). Copy number was determined using a cloned 16S rRNA gene (*Prevotella melaninogenica*) obtained from CF sputum [1, 4]. The coefficient of variation for the standards Ct value was ~1% across the range of copy number (10^3^ to 10^8^), and the assay efficiency was ≥0.86 for all plates, and the measured background was 192 ± 20 copies/reaction.

### High-throughput DNA sequencing for microbiome analysis

#### 16S amplicon library construction

Bacterial profiles were determined by broad-range amplification and sequence analysis of 16S rRNA genes [5–7]. Each DNA was amplified in triplicate along with an index-specific negative PCR control (single mastermix; termed “standard protocol” throughout). Each reaction contained 1X HotmasterMix (5Prime), 150 nM each indexed 27F, and 338R primer in a 25 μL reaction volume. Cycling conditions were 94 °C 2 min followed by 30 cycles of 94 °C 20 s, 52 °C 20 s, 65 °C 60 s. After thermal cycling, the amplicons were assessed by agarose gel electrophoresis (pooled triplicates and negative control independently) for appropriately sized bands from the DNA template and no evidence of amplification from the negative controls. If any amplification was evident in the negative control, that specimen was repeated. In addition to our standard protocol, we utilized a second modified protocol to attempt to improve sensitivity for low bacterial load samples. The additional PCR was performed exactly as in the standard protocol, but with 15nM 27F/338R primers (10% spike) that did not contain required extension for sequencing (index, linker, Illumina adapters; termed “modified protocol” throughout). All DNA extracts utilized in this study were attempted using both protocols. *PCR products were normalized using agarose gel densitometry and pooled, gel purified, and concentrated using a DNA Clean and Concentrator Kit (Zymo, Irvine, CA)*. Pooled amplicons were *quantified using Qubit Fluorometer 2.0* (Invitrogen, Carlsbad, CA). The pool was diluted to 4 nM and denatured with 0.2 N NaOH at room temperature. The denatured DNA was diluted to 20pM and spiked with 10% of the Illumina PhiX control DNA prior to loading the sequencer. Illumina paired-end sequencing was performed on the MiSeq platform using a 500 cycle version 2 reagent kit.

#### Analysis of Illumina paired-end reads

Illumina Miseq paired-end reads were aligned to human genome reference genome hg19 with bowtie2 and matching sequences discarded [8]. As previously described, paired-end sequences were sorted by sample via indexes in the paired reads with a python script [6]. Sorted paired-end sequence data were deposited in the NCBI Short Read Archive under accession number SRP133576 (https://www.ncbi.nlm.nih.gov/bioproject/PRJNA436139). The sorted paired reads were assembled using Phrap [9, 10]. Pairs that did not assemble were discarded. Assembled sequence ends were trimmed over a moving window of 5 nucleotides until average quality was met or exceeded 20. Trimmed sequences with more than 1 ambiguity or shorter than 250 nt were discarded. Potential chimeras identified with Uchime (usearch6.0.203_i86linux32) [11] using the Schloss Silva reference sequences [12] were removed from subsequent analyses. Assembled sequences were aligned and classified with SINA (1.3.0-r23838) [13] using the 418,497 bacterial sequences in Silva 115NR [14] as reference configured to yield the Silva taxonomy. Sequences with identical taxonomic assignments were grouped to produce operational taxonomic units (OTUs). This process generated 28,453,582 sequences for 168 libraries (average sequence length 312 nt; average sample size 169,367 sequences/sample; minimum 17,660; maximum 441,328). The median Good’s coverage score was ≥ 99.86% at the rarefaction point of 17,660. The software package Explicet (v2.10.5, www.explicet.org) [15] was used for visual inspection of the sequence data and analysis (rarefied alpha diversity values).

### Statistical analyses

Bacterial load was compared across the three groups, those who amplified with neither protocol, those who amplified only in the modified protocol, and those who amplified with both protocols, using a Kruskal-Wallis test. Samples where sequencing data was obtained using both the standard and the modified protocol were compared by evaluating differences in alpha diversity using paired *t*-tests. Beta diversity values between paired samples were evaluated using a one-sample *t*-test. Relative abundances for taxa were compared between protocols using generalized linear models with a negative binomial distribution, a log link, and generalized estimating equations with nested random effects (samples nested within subjects).

## Results

### Subject and samples

Sixteen subjects recruited within 24 h of intubation for mechanical ventilation support were included in this study. Each subject had tracheal aspirate samples collected daily at the first standard of care endotracheal tube clearance. DNA extractions were performed for 99 TA samples: 52 samples sequenced from both protocols, 24 samples sequenced from the modified protocol only, and 23 samples failed to amplify with either protocol. In addition, 10 samples were amplified in triplicate using each protocol to assess technical variability. Basic demographics for subjects included in this analysis are provided in Table 1.

**Table 1:** Study participant demographic information

	Overall
*n*	16
Sex = male (%)	6 (37.5)
Age mean (SD)	3.98 (5.78)
On antibiotics during study period	16 (100)

### Comparison of bacterial load

**Fig. 1:**
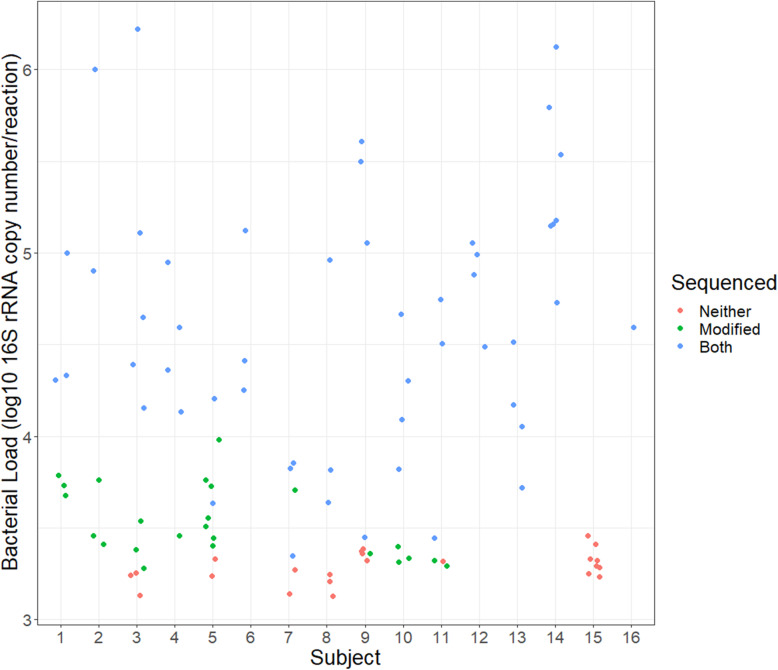
Bacterial load measurements by subject and sample. Load is shown as log 10 copy number per reaction by subject. Each point represents the average load based on triplicate measurements and is color coded to denote whether the sample amplified with neither (red), modified only (green), or both (blue) approaches. Samples with a minimum load of 1×10^4^ copies per reaction consistently amplified with the standard approach, and the minimum load for the modified approach was slightly lower at 1×10^3.3^

The median bacterial load in the 99 samples was 3.76 (range 3.13–6.22) log_10_ rRNA copies per reaction. Seven total individuals had insufficient load to obtain sequencing (failed amplification, red dots), six individuals had at least one inadequate sample, and individual 15 failed to amplify from any samples (*n*= 23, Fig. 1 red dots). The remaining samples are shown based on whether both protocols (Fig. 1 blue dots) or the modified protocol only (Fig. 1 green dots) resulted in amplification. The transition to requiring the modified protocol for amplification was not consistent between subjects, but approximately 1×10^4^ copies per reaction was required for the standard protocol to work consistently (~5x background). Not surprisingly, bacterial load was significantly higher in samples that successfully amplified with both protocols compared to those that only amplified with the modified protocol (median both 4.55 (IQR [4.15, 5.05]) log_10_ rRNA copies per reaction, median modified 3.46 (IQR [3.38, 3.71]) log_10_ rRNA copies per reaction; *p*-value <0.01). Samples that amplified with only the modified protocol had a significantly higher load than those samples that did not amplify with either protocol (median neither 3.29 (IQR [3.24, 3.33]) log_10_ rRNA copies per reaction; *p*-value< 0.01).

### Comparison of modified and standard protocols for paired samples

**Fig. 2:**
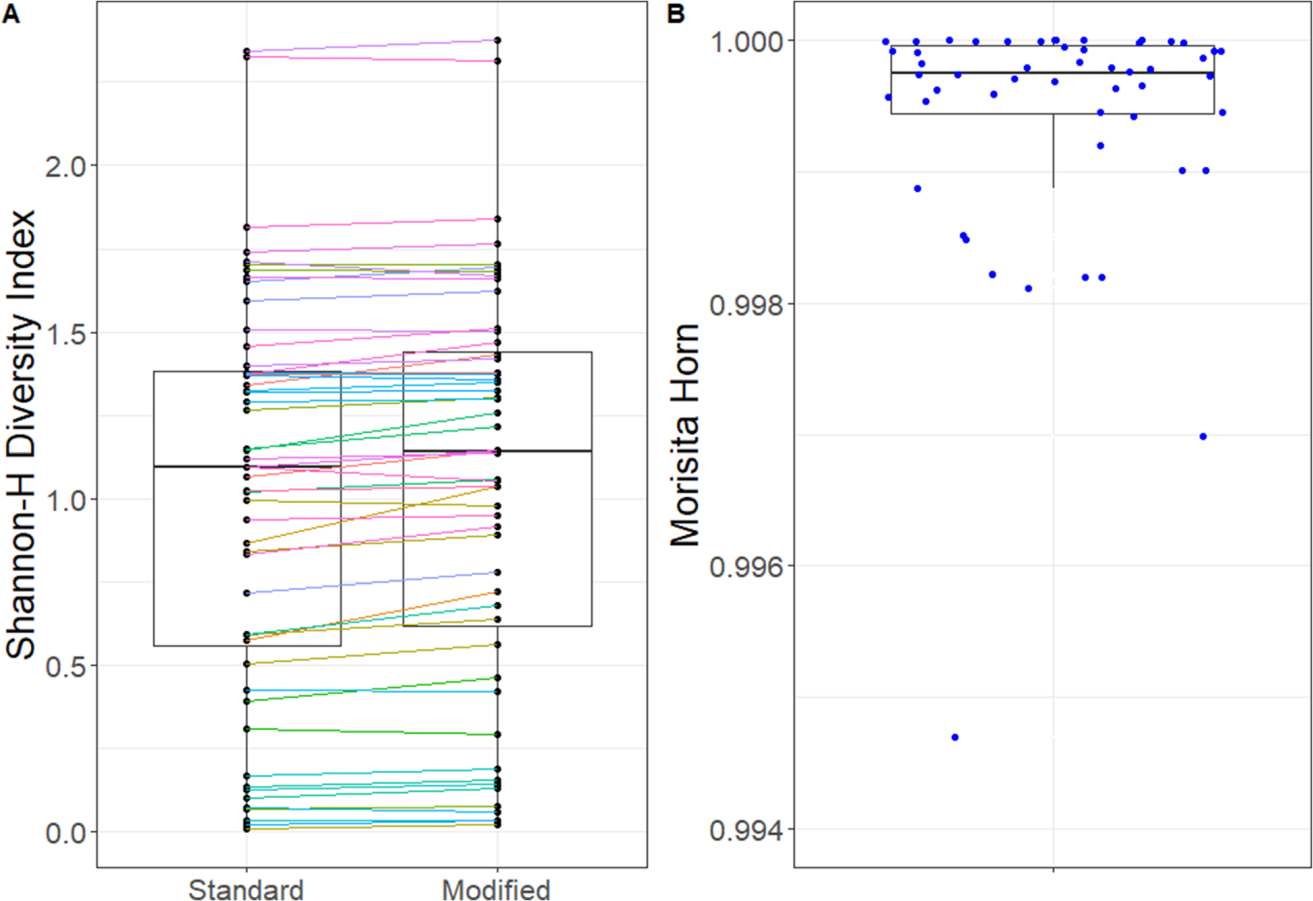
Comparison of diversity measures within and between samples. **a** Shannon diversity for each sample by approach. Paired samples (*n*=52) are connected, and the line color denotes subject (*n*=15). The box denotes median and 25th to 75th percentile values. **b** Morisita-Horn beta diversity for each paired library from each sample comparing the composition between the library from the modified and the standard method. A value of 1 indicates identical communities (composition and relative abundance). The box denotes median and 25th to 75th percentile values with whisker denoting 95%. Individual points are shown to provide the specific values for each sample. The scale is restricted to > 0.994 to improve resolution of the range of values observed

**Fig. 3:**
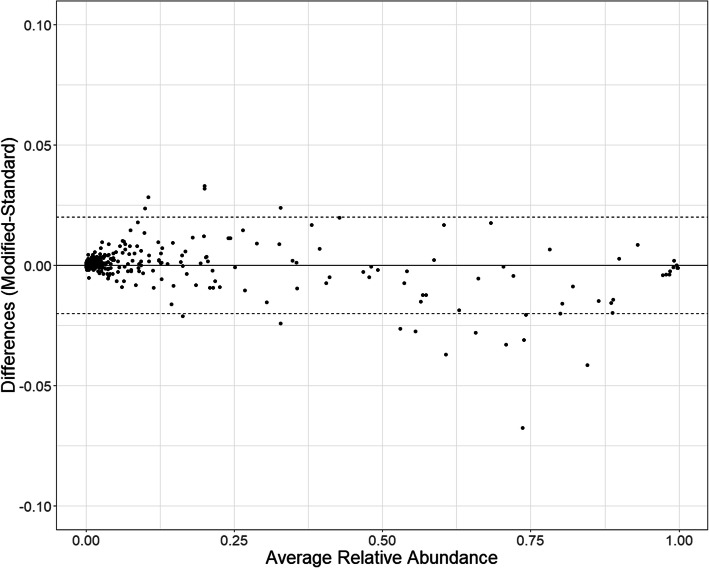
Bland-Altman plot comparing the difference in relative abundance (modified–standard RA) versus RA for each taxon from paired samples. Taxa above zero indicate higher RA in the modified approach. The black solid line indicates no difference between the modified and standard approach. The black dotted lines represent a ±2% change

Both alpha and beta diversity measures indicate that the community composition is similar between pairs in the 52 samples that amplified with both the standard and modified protocol (Fig. 2). The relative abundances for the prominent taxa are higher when using the standard protocol (more points below the line at high RA, Fig. 3). Only one taxon was identified as statistically different using the model-based approach (Table 2). *Enterobacteriaceae* had an estimated 1.9% relative abundance in the standard protocol and an estimated 2.4% relative abundance in the modified protocol. Samples from 10 subjects had a relative abundance of at least 1% for *Enterobacteriaceae*; further, only half of the samples were found to have *Enterobacteriaceae* detected (prevalence = 52%).

**Table 2:** Parameter estimates for comparing relative abundances for specific taxa using generalized linear models with a negative binomial distribution, a log link, and generalized estimating equations with nested random effects

Parameter	Estimated relative abundance standard	Estimated relative abundance modified	Pr > ||
*Streptococcus*	0.261	0.266	0.853
*Prevotella*	0.234	0.219	0.603
*Haemophilus*	0.159	0.133	0.0858
*Fusobacterium*	0.079	0.066	0.6494
*Pseudomonadales*	0.025	0.071	0.0557
*Staphylococcus*	0.042	0.050	0.5286
*Actinobacteria*	0.031	0.025	0.7982
*Enterobacteriaceae*	0.024	0.019	0.0088
*Veillonella*	0.019	0.019	0.9239
*Streptococcaceae*	0.019	0.016	0.3846

### Evaluation of samples obtained only from the modified protocol

**Fig. 4:**
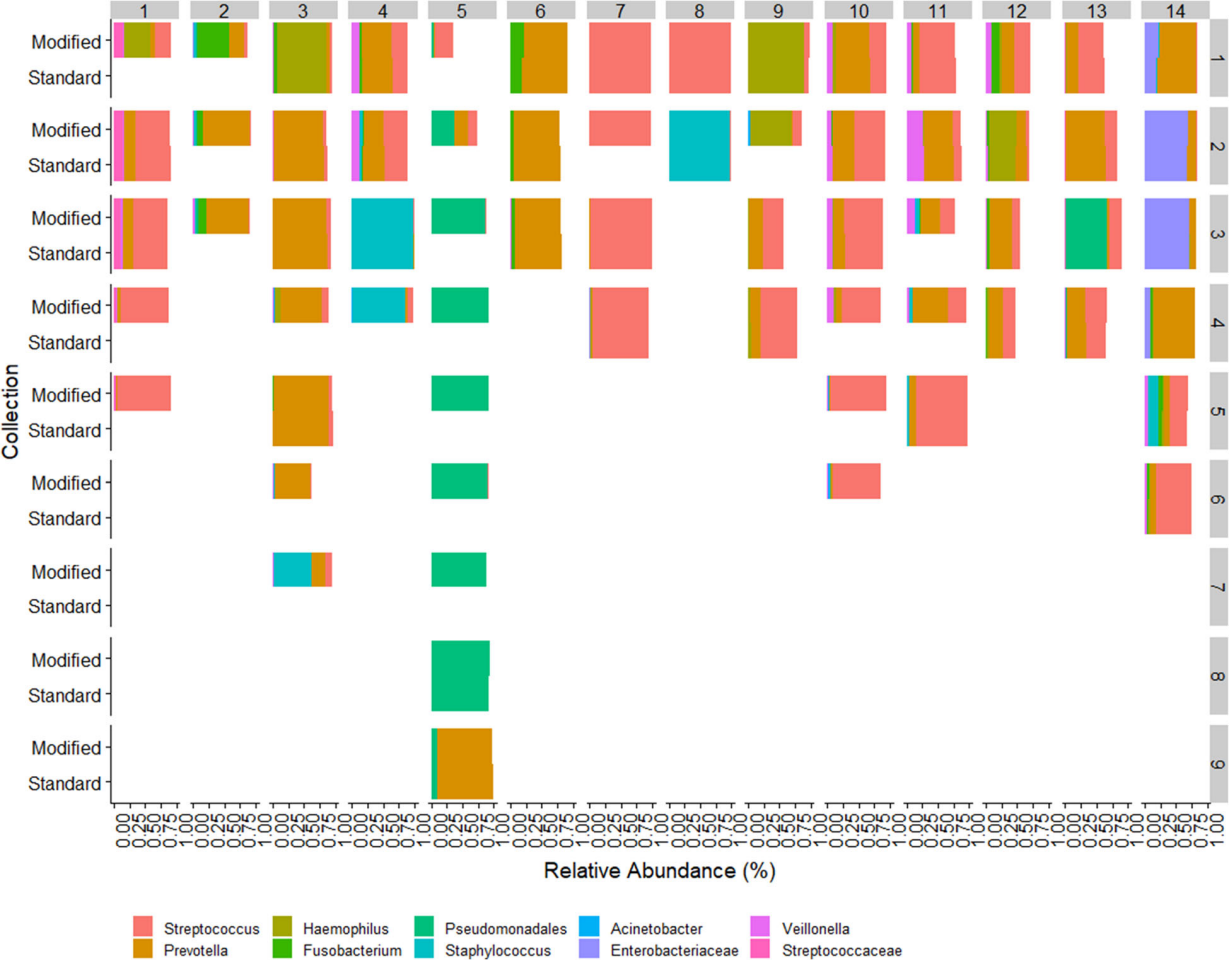
Stacked bar charts for relative abundance of the predominate taxa for each sample (row) by subject (column); subjects 15 and 16 are not displayed. Subject 15 did not amplify in any sample, and subject 16 only had one sample. Paired bar charts denote community composition with each approach. Single bar charts are shown for samples where only the modified approach provided data. The two approaches demonstrate highly similar communities

There were 24 samples from 9 subjects that only amplified using the modified protocol. Each of these 9 subjects had samples collected from other time points that did amplify using the standard protocol. Community comparisons from samples collected within subjects had consistent composition over time (Fig. 4).

### Examination of background with the modified protocol

**Fig. 5:**
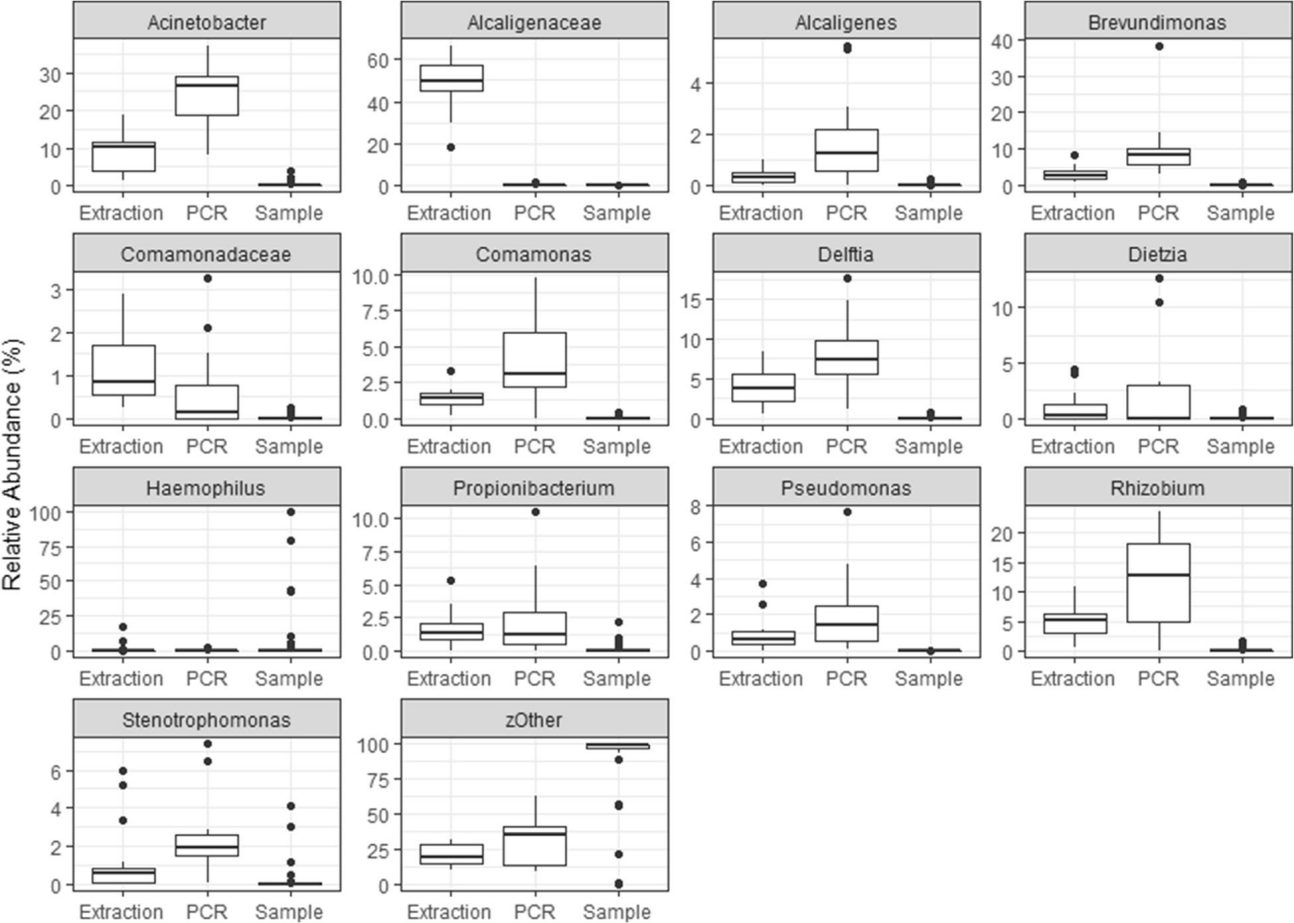
Relative abundance of prevalent taxa within control samples. Boxplots show PCR and extraction controls for each taxon identified along with the distribution of RA from clinical samples. There is limited overlap between controls and clinical samples when comparing RA of these taxa

PCR and DNA extraction controls (16 each, 32 total libraries sequenced across two amplicon pools) were evaluated using the modified method to assess the modification on background. PCR controls utilized DEPC-treated water, and the DNA extraction controls were performed using the same DEPC-treated water extracted with the Qiagen EZ1 platform (methods). There was no apparent amplification in any of these controls, and they were included in the pool with the maximum volume used for samples. The raw sequence counts obtained from control libraries was low median 3146 sequences (IQR 1732–4612). Few taxa (*n*=10; 5 exclusive to DNA extraction, 2 exclusive to PCR) were present in all 32 replicate controls, which suggest the majority of taxa observed were derived from sources other than the reagents. The number of taxa observed in control libraries increased slightly (*n*=27; 11 exclusive to DNA extraction, 7 exclusive to PCR) if prevalence in the 32 control libraries was reduced to 75% (taxa observed in ≥24 libraries). There was a single prominent taxon in the DNA extraction controls (*Alcaligenaceae*), but otherwise, the relative abundance of taxa in the control libraries was generally variable (Fig. 5).

### Reproducibility of protocols

**Fig. 6:**
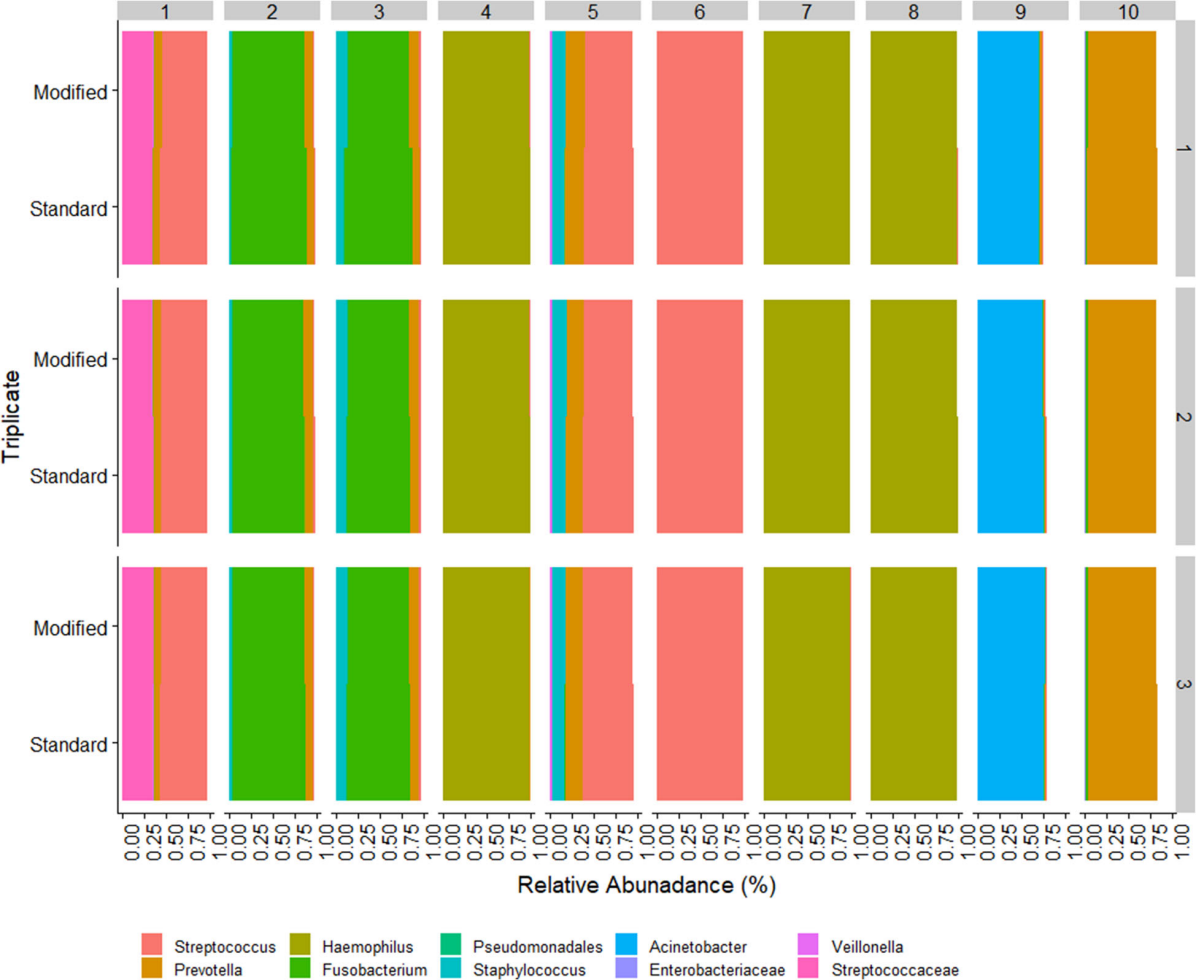
Community composition comparison for the subset of 10 sample run in triplicate. Replicate 1 was run using different indexes, and replicates 2 and 3 were run using the same indexes to confirm the sequencing primer did not influence the analysis. We see excellent agreement both within and between approaches

Ten samples with adequate load were selected to amplify with both protocols using 3 independent PCRs (four additional libraries for each DNA). Figure 6 shows the relative abundance at which taxa were consistently detected in all three replicates for both protocols. For the standard and modified protocols, the relative abundance at which taxa were repeatedly detected in all replicates was 0.01% and 0.027%, respectively (Figure S1).

## Discussion

The modified protocol provided sequence data from 31.5% more samples compared to the standard protocol (52 vs. 76 of 99). For samples that produced sequencing results using both protocols, the bacterial communities were similar by alpha and beta diversity measures, but some variation was observed in the RA of more abundant taxa (≤7% RA), While *Enterobacteriaceae* RA was significantly different between protocols, this taxon was only observed in one individual making it challenging to generalize. Further, the absolute change in estimated RA was small (0.5%) and would not alter biological inferences, which is the primary factor for most analyses. Both protocols had similar reproducibility and the lower limits of RA where all replicates amplified were much lower than typical cutoffs used when evaluating specific taxa.

There is some controversy regarding the impact of the additional primer sequence on the bacterial community composition observed depending on the experimental design. Berry et al. [16] proposed an approach, termed 2-step, where standard primers were used in the first round of PCR to amplify sample DNAs, and then, a second round of PCR was performed to add the sequence platform-required oligonucleotides using the amplicons from the first round as template. They observed statistical differences in alpha diversity (Bray-Curtis, unweighted UniFrac). However, the prominent taxa identified did not change suggesting the alterations would not greatly impact biological conclusions. A limitation of this study was the absence of replication to determine how much influence biological and technical replicates introduce into the analysis. Our protocol modification emulated the approach by Berry, but without running two separate PCR reactions. The experimental modification was to spike in 16S rRNA gene-specific primers at 10% of the standard primer concentration used for the longer sequencer-specific indexing primers. The rationale was the short primers would initiate PCR more efficiently in early cycles and increase the concentration of the 16S rRNA target sequences for the longer modified primers sufficiently to obtain adequate amplification for reliable sequencing.

The impact from background is a concern when attempting to amplify low load samples [3]. We demonstrated that the modification did not introduce significant background into the analysis, which is largely due to our very conservative approach to PCR amplification (mainly limited cycle number). We eliminated 23% of the samples prior to sequencing due to multiple independent pieces of evidence that inadequate bacteria were present (low TBL; no amplification apparent from either PCR protocols) for reliable data collection (essentially QNS, quantity not sufficient). This is in contrast to published approaches that suggest obtaining reliable sequence data from all samples is feasible. We agree that it is possible to obtain sequence data in all cases if adequate protocol deviation is tolerated (particularly excessive PCR cycles) but question the utility given the difficulty in attributing the source of sequences derived from this type of experiment. The high level of variation observed in our background experiments suggests that reagents were a minor source of background at lower PCR cycle numbers, and the most likely source of significant portions of the background sequence is due to the samples amplified in parallel [3]. The DNA template added to adjacent wells represent the largest potential exposure, and that is why we utilize a negative PCR control for all samples (in contrast to plate level controls). This control does not guarantee that transfer between wells does not occur but does allow us to assess the magnitude of potential cross-contamination and take appropriate measures to mitigate in cases with clear contamination.

We observed a non-significant increase in alpha diversity, which may raise concerns about background. The increased efficiency of the PCR should yield an increase in richness particularly due to better sampling of rare taxa; improved efficiency simply provides access further out the rank abundance curve independent of the source of DNA. Our replicate data demonstrates excellent agreement within technical replicates to RA levels much lower than that typically used of specific taxa (~0.005% RA versus >1% RA for specific taxa). Pragmatically, interpretation of low RA observations is challenging independent of the source (sample or background). These data demonstrate very good agreement for typically used metrics and provided data for 24 additional samples (51% of samples that failed to amplify initially) without major impacts to data integrity.

These data address several critical questions. First, inclusion of non-adapter-linked primers in the primary PCR could inhibit the ability to obtain sequence data. Amplicons without sequencing adapters are likely to co-purify with the target amplicons during processing steps required for sequencing. We did appear to lose some cluster density with the modified protocol, but with limited sequencing runs, it is difficult to examine this critically. Based on the minimum library size (>17,000 sequences) and Good’s coverage >99.86%, this was not an issue. Second, the anticipated mode of action for the modified protocol was to increase efficiency of early rounds of PCR, which should improve sensitivity. We had observed a shift of approximately three cycles in Ct when using the different primers (with and without sequencing adapters) in simple qPCR experiments (data not shown), which suggested about a 10-fold loss of efficiency for the primers with sequencing adaptors. These observations were the basis for the experiment described here, which does support the expected mode of action for the experimental intervention. Third, it was not known if increased efficiency in early rounds of PCR would impact the community composition observed in the experiment. Variation in early amplification is known to impact amplicon composition and is the reason we run multiple independent reactions and pool them to mitigate any potential impact from this known issue [17]. The high beta diversity values observed indicate very good agreement between the two approaches. Further, the technical replicates demonstrated highly reproducible results from both PCR protocols.

## Conclusion

We show that both protocols identify similar communities with typical airway composition, and the technical variability of both protocols was very low. The modified protocol was successful in amplifying more samples compared to the standard protocol especially for those samples with intermediate bacterial load. This is likely due to the improved efficiency of PCR using non-fusion primers. Therefore, our modified amplification protocol provides a viable alternative for increasing the sensitivity of bacterial community analysis in specimens with intermediate bacterial load without impacting data integrity.

## Supplementary Information

**Additional file 1: Figure S1.** Lower limit of detection from triplicates. The y axis is the number of triplicates in which a taxon was detected and the x-axis is the average RA across the triplicates for each taxon and sample. Points are jittered along the y-axis to better display the spread of points. Black dots correspond to the modified approach and blue dots to the standard approach. A) displays the lower range of RA values and B) displays the full range.

## Data Availability

Sorted paired-end sequence data were deposited in the NCBI Short Read Archive under accession number SRP133576 (https://www.ncbi.nlm.nih.gov/bioproject/PRJNA436139).
