# Supplementary material for: Modified PCR protocol to increase sensitivity for determination of bacterial community composition
Source: Microbiome. 2021 Apr 13;9:90. doi: 10.1186/s40168-020-00958-y (PMC8045227; doi:10.1186/s40168-020-00958-y)

Supplemental Figures:

Figure S1: Lower limit of detection from triplicates. The y axis is the number of triplicates in which a taxon was detected and the x-axis is the average RA across the triplicates for each taxon and sample. Points are jittered along the y-axis to better display the spread of points. Black dots correspond to the modified approach and blue dots to the standard approach. A) displays the lower range of RA values and B) displays the full range.


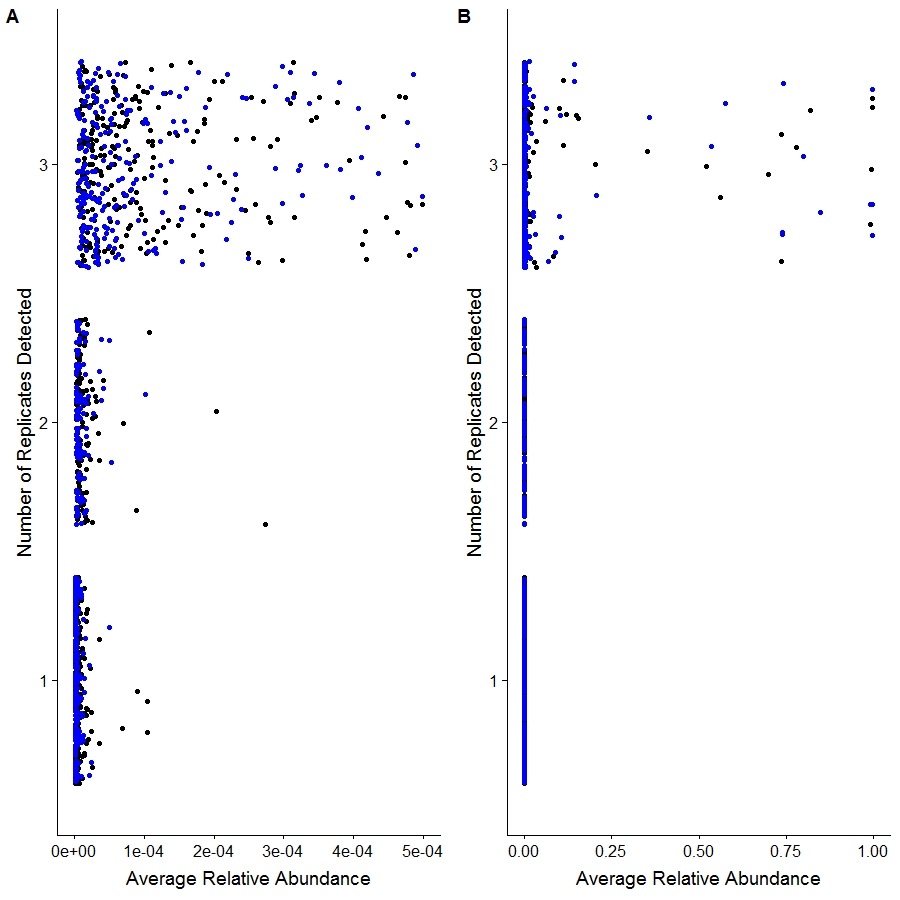

Supplement: Supplementary file 2 — Additional file 1: Figure S1. Lower limit of detection from triplicates. The y axis is the number of triplicates in which a taxon was detected and the x-axis is the average RA across the triplicates for each taxon and sample. Points are jittered along the y-axis to better display the spread of points. Black dots correspond to the modified approach and blue dots to the standard approach. A) displays the lower range of RA values and B) displays the full range. [file 40168_2020_958_MOESM2_ESM.docx]
